# Combined balance and plyometric training enhances knee function, but not proprioception of elite male badminton players: A pilot randomized controlled study

**DOI:** 10.3389/fpsyg.2022.947877

**Published:** 2022-08-09

**Authors:** Limingfei Zhou, Wangcheng Gong, Shixian Wang, Zhenxiang Guo, Meng Liu, Samuel Chuang, Dapeng Bao, Junhong Zhou

**Affiliations:** ^1^School of Strength and Conditioning Training, Beijing Sport University, Beijing, China; ^2^School of Physical Education, Jiujiang University, Jiujiang, China; ^3^Sports Coaching College, Beijing Sport University, Beijing, China; ^4^Department of Physical Education, Nanjing University of Aeronautics and Astronautics, Nanjing, China; ^5^Human Biology Major, University of California, San Diego, San Diego, CA, United States; ^6^China Institute of Sport and Health Science, Beijing Sport University, Beijing, China; ^7^Hebrew Senior Life Hinda and Arthur Marcus Institute for Aging Research, Harvard Medical School, Boston, MA, United States

**Keywords:** plyometric exercise, physical conditioning, knee, postural balance, badminton

## Abstract

**Objectives:**

To investigate the effect of combined balance and plyometric training on knee function and proprioception of elite badminton athletes.

**Methods:**

Sixteen elite male badminton players (age: 20.5 ± 1.1 years, height: 177.8 ± 5.1 cm, weight: 68.1 ± 7.2 kg, and training experience: 11.4 ± 1.4 years) volunteered to participate and were randomly assigned to a combined balance and plyometric training (CT) (*n* = 8) and plyometric (PT) group (*n* = 8). The CT group performed balance combined with plyometric training three times a week over 6 weeks (40 min of plyometrics and 20 min of balance training); while the PT group undertook only plyometric training for the same period (3–4 sets × 8–12 reps for each exercise). Both groups had the same technical training of badminton.

**Results:**

The knee function and proprioception were assessed at baseline and after the intervention by measuring the performance of single-legged hop tests (LSI_O_, LSI_T_, LSI_C_, LSI_S_), standing postural sway (COP_AP_, COP_ML_), and LSI of dominant leg and non-dominant leg. The results showed that as compared to PT, CT induced significantly greater improvements in LSI_T_ and LSI_S_ (*p* < 0.001) and significant greater percent increase in N_AP_ (*p* = 0.011). The changes in LSI_O_, LSI_C_, D_AP_, N_AP_, LSI_AP_, D_ML_, N_ML_, and LSI_ML_ induced by CT did not differ from that induced by PT (*p* > 0.213).

**Conclusion:**

In elite badminton players, intervention using CT holds great promise to augment the benefits for knee function compared to the intervention using PT only, and at the same time, with at least comparable benefits for proprioception. Future studies are needed to examine and confirm the results of this study.

## Introduction

Badminton is considered as one of the fastest racket sports (Phomsoupha and Laffaye, 2015; Abdullahi and Coetzee, 2017; Gómez et al., 2021), requiring frequent quick starts, stops, lunges, and changes of direction (Shariff et al., 2009; Hong et al., 2014). These high-intensity and quick movements and reactions in badminton significantly increased the risks of injuries to lower extremities, such as anterior cruciate ligament (ACL) injury of knees, one type of injury that frequently occurs in badminton athletes (Kimura et al., 2012; Alikhani et al., 2019; Zhao and Gu, 2019). In addition, badminton matches require players to adjust their body position continuously throughout the game, in which their capacity of dynamic balance to maintain their center of gravity within the base of support to react to the moving shuttlecock (Faude et al., 2007; Chang et al., 2013). Strategies aiming to improve knee function and proprioception are thus beneficial for the on-court performance of badminton players and can help reduce the risk of injuries (White et al., 2013).

One such strategy is plyometric training (PT), of which the goal is to improve lower limb strength, knee function (e.g., isokinetic muscle strength test and single-legged hop tests), and movement pattern of landing (e.g., depth jump and continuous jump) (Hewett et al., 2010) by shortening muscle eccentric-concentric contraction cycle [also termed as a stretch-shortening cycle (SSC)] (Markovic and Mikulic, 2010; Ramírez-delaCruz et al., 2022). Studies have shown that PT can enhance the sport performance of athletes, such as strength (Asadi, 2012), running economy (do Carmo et al., 2022), agility (Maciejczyk et al., 2021), and sprint ability (Markovic and Mikulic, 2010), as well as reducing ACL injury risk. Alikhani et al. (2019) observed that, for example, 6-week PT significantly improved dynamic balance and knee proprioception in female badminton players by enhancing their functional adaptations and neural recruitment of motor units that activate appropriate muscles before landing and the proprioceptive inputs.

More recently, studies have emerged to combine PT with other training programs and observed that this combined training (CT) can significantly augment the benefits of using PT only for knee function and proprioception (Nessler et al., 2017). These kinds of CT can simultaneously enhance multiple aspects contributing to knee function and proprioception, target reflexive response and proprioception, and help adjust the body positioning while landing with correct knee and hip position (Hewett et al., 2006). The study of Wedderkopp et al. (2003) for example, showed that combined training of balance and strength is of great promise to reduce the incidence of injuries in young female handball players. Additionally, several of our previous studies showed that combined training of balance and PT significantly improved the capacity to adjust the direction of movements and dynamic balance, which are essential for athletic performance and the prevention of injury risk (Guo et al., 2021; Lu et al., 2022). These findings suggest that compared to PT only, CT may induce greater benefits for athletes of badminton by simultaneously augmenting their knee function and proprioception, which, however, have not been examined.

Therefore, this pilot randomized controlled study aims to characterize the effects of a 6-week CT with balance training and plyometric training on knee function and proprioception in a group of elite badminton players and examine if this kind of CT can induce greater benefits as compared to PT. Participants completed the CT and PT of the same training protocol as validated in our previous studies (Guo et al., 2021; Lu et al., 2022). Specifically, we hypothesize that the CT protocol would induce a significant increase in performance (e.g., single-legged hop tests and center of pressure) pertaining to knee function and proprioception compared to PT.

## Materials and methods

### Participants

Sixteen healthy elite male badminton players were recruited in the study. The inclusion criteria were: (1) Elite players who had won the top four of national youth games and provincial games, or higher-level games; (2) The dominant arm or leg is the right side; and (3) the ability and willingness to complete the 6-week programs of tests and intervention. The exclusion criteria were: (1) Participants had ACL, hamstring, meniscus, ankle, or any other lower-extremity injuries during the last 3 years, and (2) Limb Symmetry Index (LSI) of single-legged hop tests were < 85%. Eight players entered the quarterfinalists of national youth games and the rest entered the finals at the provincial level. All the participants were from the same club, all right-handed, and undertook three training sessions per week each of which consisted of 2–3 h of technical and physical training sections. The study protocol was approved by the Research Ethics Committee of Beijing Sport University (Approval number: 2020008H), and all procedures were conducted in accordance with the Declaration of Helsinki. Before data collection, the participants were informed about the benefits and possible risks associated with the study, and the participants provided written informed consent to participate.

### Procedures

All experimental training programs were conducted along with a weekly technical training routine. Participants were randomized into the group of CT (*n* = 8) and the group of PT (*n* = 8) (Table 1). Before the initiation of the study, all participants completed a 2-week familiarization (three sessions per week) with the same training protocols as used in the following intervention in this study. During the intervention period, participants in CT group completed the intervention that combining the balance training and PT. Specifically, they completed three sessions of CT per week for 6 weeks (i.e., 18 sessions). Within each session of CT, they were asked to complete 40 min of PT (e.g., depth jump and lateral barrier jump) and then 20 min of balance training that was performed on an unstable support (e.g., BOSU ball, Swiss ball, and Balance pad). In PT group, participants also completed three sessions of PT per week for 6 weeks. To ensure a similar training load between CT and PT group, in each session the participants in PT group first completed 40 min of PT and then 20 min of balance training that was the same as CT group, but on the stable support (i.e., solid floor). The recovery period of 24–48 h was provided between each training session. The details of the protocols of balance training and PT were included in Supplementary Material.

**TABLE 1:** The descriptive characteristics of the participants.

	Age (years)	Height (cm)	Weight (kg)	Training experience (years)
**PT (*n* = 8)**	19.13 ± 2.23	179.13 ± 6.06	69.88 ± 8.94	10.63 ± 1.06
**CT (*n* = 8)**	20.50 ± 1.07	177.75 ± 5.06	68.13 ± 7.22	11.38 ± 1.41

**FIGURE 1:**
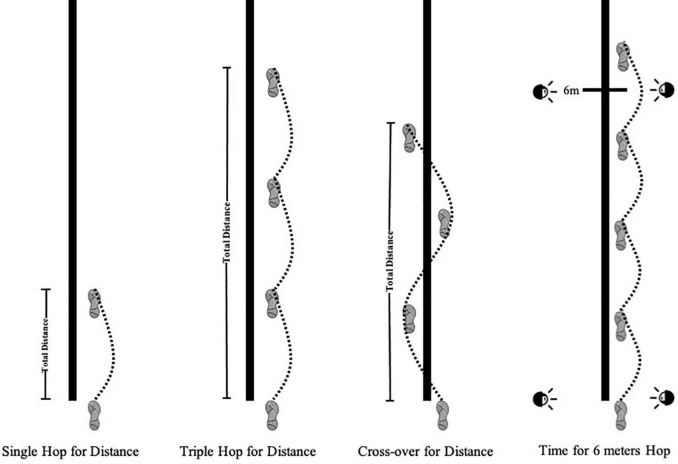
Single-legged hop tests.

Single-legged hop tests hold potential as predictive factors of knee function in individuals to evaluate the risk of ACL injury and discriminate between those individuals who return to previous activity level after ACL injury or reconstruction (Figure 1; Noyes et al., 1991). The single hop for distance, triple hop for distance, cross-over hop for distance and timed for 6 meters hop were measured, respectively. Smart Speed device (Fusion Sport, Coopers Plains, Australia) was set for Time for six meters hop to record the time. After hopping, participants needed to stand with a single leg for 2 s to make results effective. Participants were asked to jump three times every leg in each test, and the longest distance and the shortest time in the three tests were taken as the final data when the four tests’ lower LSI was calculated. LSI was counted as the ratio between the non-dominant leg and dominant leg while timed for 6 meters hop was calculated by dominant and non-dominant in the division. Four kinds of LSI were defined as LSI_O_ (Single Hop for Distance), LSI_T_ (Triple Hop for distance), LSI_C_ (Cross-over for distance), LSI_S_ (Time for 6 meters Hop) in this study. When LSI ≥ 85%, there is no risk of ACL injury; and LSI < 85%, ACL is at risk of injury (Noyes et al., 1991).

The proprioception test was used to assess the balance performance of players by the center of pressure (COP) (Sell, 2012), which evaluate the proprioception of players to observe the injury risk of knee or ankle (Thompson et al., 2017). Participants stood on an in-ground force plate (Kistler 9281CA, KISTLER, Winterthur, Switzerland, 1,000 Hz) and stood with a dominant or non-dominant leg for 10 s. MATLAB software (r2014b, MathWorks, Natick, Massachusetts, United States) was used to calculate COP. All data were smoothed through low-pass filtering, and the truncation frequency was set to 13.33 Hz.

COP was calculated from the time series within 10 s after standing, and anterior-posterior (AP) displacement difference, and medial-lateral (ML) displacement difference of both legs (D_AP_, N_AP_, D_ML_, N_ML_) (Ziv and Lidor, 2010). X_T_ and Y_T_ are the anterior-posterior (AP), medial-lateral (ML) displacements at t seconds and the value of t are 1–10 s. LSI of COP was counted as the ratio between non-dominant leg and dominant leg while timed for 6 meters hop was calculated by dominant and non-dominant in the division.


(1) $$\text{AP}=\sum_{1}^{10}{\left(X_{\text{t}}-\bar{X}\right)}^{2}$$

(2) $$\text{ML}=\sum_{1}^{10}{\left(Y_{\text{t}}-\bar{Y}\right)}^{2}$$

### Statistical analysis

Experimental data were processed by IBM SPSS statistical software package (version 25.0, IBM, Chicago, IL, United States). All data were presented as means and SD. The level of significance was set at *p* < 0.05 for all tests. To examine the effects of the combined training on the performance of single-legged hop tests and proprioception tests, we firstly performed two-way repeated-measure ANOVA (group × time). The dependent variable for each model was LSI_O_, LSI_T_, LSI_C_, LSI_S_, D-(AP, ML, LSI), and N-(AP, ML, LSI). The model factors were group, time, and their interaction. When a significant interaction was observed, LSD post-hoc correction was performed to identify the location of the significance. Secondly, we examined the effects of CT on the performance within each group, and the percent changes from pre- to post-intervention between CT and PT by using separate one-way ANOVA models. The model factor was time. Partial η^2^ was used to assess the effect size (ES) where the significance was observed, with its strength being interpreted as the following: < 0.06 as small, < 0.14 as moderate, and ≥ 0.14 as large (Cohen, 2013).

## Results

The primary two-way repeated-measures ANOVA models showed significant interactions between group and time on LSI_T_ (*p* = 0.008) and LSI_S_ (*p* = 0.019) but not on LSI_O_ and LSI_C_ (*p* > 0.507). The *post-hoc* analysis revealed that the LSI_T_ [*F*_(1, 28)_ = 7.535, *p* = 0.010, partial η^2^ = 0.212] and LSI_S_ [*F*_(1, 28)_ = 14.402, *p* = 0.001, partial η^2^ = 0.340] in CT group were significant greater after the intervention as compared to all the other pre- and post-interventions (Table 2).

**TABLE 2:** The assessment results for CT group and PT group before and after the 6-week training.

									The effects of time,		
									group and their		
	CT (*N* = 8)				PT (*N* = 8)				interaction (*p*-value)		
				Partial				Partial			Time ×
Variable	Pre	Post	△	η2	Pre	Post	△	η2	Time	Group	group
LSI_O_ (%)	90.74 ± 4.15	95.43 ± 3.21*	5.33 ± 5.25	0.208	88.25 ± 2.51	91.97 ± 3.74*	4.31 ± 5.86	0.141	0.002	0.022	0.691
LSI_T_ (%)	87.13 ± 1.71	96.55 ± 4.46*#	10.8 ± 5.63#	0.548	89.30 ± 3.72	92.16 ± 2.78*	3.30 ± 3.66	0.100	<0.001	0.340	0.008
LSI_C_ (%)	91.04 ± 4.56	94.28 ± 2.21	3.74 ± 4.73	0.079	91.77 ± 4.78	93.02 ± 4.57	1.52 ± 5.55	0.013	0.138	0.857	0.507
LSI_S_ (%)	87.51 ± 1.63	99.68 ± 2.69*#	13.93 ± 2.90#	0.813	87.20 ± 1.46	95.49 ± 2.73*	9.55 ± 3.87	0.668	<0.001	0.007	0.019
D_AP_ (cm)	90.79 ± 7.67	71.50 ± 10.31*	20.88 ± 12.38	0.327	93.33 ± 14.36	79.58 ± 8.11*	13.80 ± 9.07	0.198	<0.001	0.167	0.460
N_AP_ (cm)	102.67 ± 8.33	72.20 ± 10.81*	29.75 ± 7.52#	0.585	103.95 ± 11.18	83.72 ± 8.05*	19.11 ± 7.09	0.383	<0.001	0.072	0.146
LSI_AP_ (%)	89.28 ± 3.79	98.15 ± 7.28*	11.27 ± 11.49	0.197	90.99 ± 5.21	95.37 ± 9.45	6.84 ± 10.48	0.056	0.006	0.826	0.472
D_ML_ (cm)	88.37 ± 8.22	80.44 ± 10.60	9.00 ± 8.09	0.039	89.54 ± 11.94	82.11 ± 11.60	8.29 ± 4.59	0.031	0.165	0.485	0.933
N_ML_ (cm)	99.65 ± 8.77	80.76 ± 7.22*	18.75 ± 6.53	0.174	102.08 ± 15.85	87.81 ± 15.60*	13.87 ± 8.91	0.036	<0.001	0.233	0.605
LSI_ML_ (%)	88.77 ± 4.68	100.19 ± 15.09*	12.47 ± 12.01	0.139	88.08 ± 4.77	94.66 ± 12.03	7.55 ± 13.32	0.050	0.019	0.371	0.508

LSI, limb symmetry index; D, dominant leg; N, non-dominant leg; △, percentage changes between pre- and post- test; Partial η^2^, effect size of between-group comparisons.

*Statistically significant difference between pre- and post-test, p < 0.05.

^#^Statistically significant difference between CT group and PT group, p < 0.05.

For LSI_O_ and LSI_C_, the secondary one-way ANOVA models showed that the LSI_O_ was significantly improved as compared to pre-intervention within either CT and PT group (*p* < 0.048), while no significant effect and percent change in LSI_C_ of both groups was observed (*p* > 0.719). Additionally, the percent changes in LSI_T_ (*p* = 0.007) and LSI_S_ (*p* = 0.023) within the CT group was significantly improved compared to PT group, indicating greater benefits was induced by CT.

The primary two-way repeated-measures ANOVA models revealed no significant interaction of group and time on D_AP_ (*p* = 0.460), N_AP_ (*p* = 0.146), LSI_AP_ (*p* = 0.472), D_ML_ (*p* = 0.933), N_ML_ (*p* = 0.605) or LSI_ML_ (*p* = 0.508). Only significant effects of time on D_AP_, LSI_AP_, N_AP_, N_ML_, and LSI_ML_ (but not on D_ML_) were observed (D_AP_: *p* < 0.001; N_AP_: *p* < 0.001; LSI_AP_: *p* = 0.006; N_ML_: *p* < 0.001; LSI_ML_: *p* = 0.019).

Secondarily, one-way ANOVA models showed that compared to pre-intervention, D_AP_, D_ML_, N_AP_, and N_ML_ were significantly decreased after the intervention within both CT and PT groups (*p* < 0.014) and within CT, LSI_AP_, and LSI_ML_ were significantly improved after intervention (LSI_AP_: *p* = 0.014; LSI_ML_: *p* = 0.033), while no significant difference was observed within PT group. Additionally, the percent changes in N_AP_ within the CT group were significantly improved compared to PT group (*p* = 0.011), indicating greater benefits was induced by CT.

## Discussion

This pilot study demonstrated that combined training (CT) of balance and plyometric training (PT) is of great promise to enhance knee function and proprioception. Multiple aspects of knee function and proprioception (e.g., LSI_O_, D_AP_, LSI_AP_, N_ML_, LSI_ML_) were improved after the intervention. Though as compared PT, significantly greater benefits of CT were observed only for LSI_T_ and LSI_S_, the results of this pilot study suggest that CT can at least induce comparable benefits to that by PT-only intervention, providing critical knowledge of study design, sample size estimation for future large-scale randomized trials.

We observed that compared to PT, CT significantly improved LSI_T_ and LSIs but not on LSI_C_ and LSI_O_. LSI_T_, and LSIs mainly reflect knee functions and limb symmetry for the continuous hopping process of the stretch-shortening cycle (SSC) (Fitzgerald et al., 2000). For example, Zwolski et al. (2016) has shown that LSI_S_ can be used to identify dynamic stability of knees in ACL injury rehabilitation.

LSI_O_ only reflects muscle strength of single-leg and limb symmetry between legs, and LSI_C_ reflects the capacity of imposing forces in frontal and transverse planes with multiple hops in the sagittal plane, which is a more technique-demanding condition (Logerstedt et al., 2012). The traditional PT focuses only on enhancing the musculoskeletal function and consolidating the movement pattern of landing, which may thus particularly benefit the LSI_C_ and LSI_O_. The balance training of CT protocol focuses on multiple abilities of balance and stability including the posterior thigh muscles, the abdominal muscles, and the hip muscles, especially improving the stability of ankle during the process of landing and take-off (Hewett et al., 2010). Hariri and Sadeghi (2018) found that it was necessary to increase the speed of the knee in order to achieve maximum foot speed toward the target in the performance of the Karate. Improving muscle performance and joint movement is thus of great importance in quick movement during badminton matches, especially in enhancing knee function. CT intervention thus can simultaneously target lower limb strength, knee function, and movement pattern of landing, and can improve both power and agility of badminton players (Fischetti et al., 2018; Guo et al., 2021). It may thus induce significantly greater improvements specifically in LSI_T_ and LSIs (but not LSI_C_ and LSI_O_) by targeting SSC and dynamic stability of knees, suggesting particular benefits from CT on dynamic stability of knees.

However, as compared to PT, no significant improvements in proprioception as assessed by COP outcomes induced by CT were observed, suggesting CT induced benefits for proprioception which are only comparable to PT. The proprioception and stability of postural control require the integration of the visual, vestibular information and coordination across those systems, among which the strength of the lower limb is extremely important (Wrisley and Stephens, 2011; Zhao and Gu, 2019). Alikhani et al., observed that 6-week of PT improved dynamic balance and knee proprioception in female badminton players (Alikhani et al., 2019). Additionally, Nepocatych et al. (2018) observed that balance training can increase ankle stability by augmenting neuromuscular function and proprioception as well as the dynamic balance by optimizing the postural control system to more efficiently utilize multiple kinds of sensory inputs (e.g., visual, proprioceptive) (Söderman et al., 2000; Guo et al., 2021; Lu et al., 2022). However, no significant difference in COP outcomes between CT and PT is observed here, though a greater percentage change in these outcomes was induced by CT compared to PT, which thus needs to be further explored in future studies.

Meanwhile, one potential reason for non-significant results may be the ceiling effects that only elite professional players were included, and using PT may be sufficient to induce maximum improvements in proprioception. Taken together, our results show that CT may uniquely augment knee function, suggesting that this type of CT would be a helpful strategy that can be included in the training routines of elite badminton players with adaptive protocols to maximize the benefits for athletes (e.g., using balance training as a passive rest method between different sets of exercises).

Several limitations of this pilot study should be noted. First, only sixteen male elite badminton players were included, so the generalizability of the study findings was limited. Studies with larger sample size and consisting of both male and female participants, as well as of other cohorts (e.g., adolescent players), are highly demanded to further examine and confirm the observations in this study. Second, we here only examined the short-term/immediate effects of CT, studies consisting of longer-term follow-up assessments and the record of athletic injury incidence are needed to examine how such benefits can sustain and if CT can help reduce the incidence of injuries in athletes. Additionally, we here implemented the CT following our previous work, it is worthwhile to examine the effects of other types of CT protocol, and to explore the appropriate training intensity and number of sessions that can help maximize the benefits of CT. Third, future studies can implement assessments which are more closely linked to on-court performance (e.g., badminton-field reaction test and three-dimensional motion capture technique) to examine the benefits of CT for badminton performance, in addition to the knee function. Additionally, future studies are needed to examine the effects of CT on other psychological and physiological aspects related to badminton, such as cognitive function, and mood, to comprehensively assess the benefits of CT on this cohort.

## Conclusion

This pilot study showed that balance training combined with plyometric training program may induce a significant effect on knee function and at least comparable positive effects on proprioception of elite badminton players as compared to plyometric training only. The knowledge obtained from this pilot study will ultimately help inform the design of future larger-scale studies to confirm the findings here.

## Data availability statement

The raw data supporting the conclusions of this article will be made available by the authors, without undue reservation.

## Ethics statement

The study protocol was approved by the Research Ethics Committee of Beijing Sport University (Approval number: 2020008H), and all procedures were conducted in accordance with the Declaration of Helsinki. The patients/participants provided their written informed consent to participate in this study.

## Author contributions

LZ, WG, SW, ZG, and DB: research concept and study design. LZ, WG, SC, DB, and JZ: literature review and writing of the manuscript. LZ, WG, and DB: conceptualization and methodology. SW, ZG, and ML: formal analysis, investigation, and resources. ZG, ML, and JZ: data collection, data analysis and interpretation, and statistical analyses. LZ and WG: writing—original draft preparation. LZ, SC, DB and JZ: writing—review and editing. All authors have read and agreed to the published version of the manuscript.
