# Supplementary material for: Combined balance and plyometric training enhances knee function, but not proprioception of elite male badminton players: A pilot randomized controlled study
Source: Front Psychol. 2022 Aug 9;13:947877. doi: 10.3389/fpsyg.2022.947877 (PMC9396213; doi:10.3389/fpsyg.2022.947877)
Supplement: Supplementary file 1 [file Table_1.docx]

Supplementary Material

**Table S1.** The balance training program for balance-plyometric (combined training) (CT) group and plyometric training (PT) group.

| Exercises | The first stage (1-2 weeks) | The second stage (3-4 weeks) | The third stage (5-6 weeks) |
| --- | --- | --- | --- |
| Stand on the balance board exercise | Static standing on the board with two legs  (3 sets: 30 s/set) | Static standing on the board with two legs and eyes closed  (3 sets: 30 s/set) | Squat on the plate with eyes closed  (3 sets: 10 reps/set) |
| Supine straight leg bridge on Swiss Ball | Isometric supine straight leg bridge on Swiss Ball  (3 sets: 30 s/set) | Isometric supine single-leg bending bridge on Swiss Ball  (3 sets: 30 s/set) | Dynamic supine single-leg bending bridge on Swiss  (3 sets: 10 reps/set) |
| Side-plank with inflated balance disc | Side-plank with inflated balance disc with elbow  (3 sets: 30 s/set) | Side-plank with inflated balance disc and the non-supporting leg stretches backward  (3 sets: 10 reps/set) | Side-plank with inflated balance disc and the non-supporting leg stretches backward with elastic band  (3 sets: 10 reps/set) |
| Lunge squat on BOSU ball | Lunge squat on BOSU ball  (3 sets: 10 reps/leg/set) | Lunge squat on BOSU ball and inflated balance disc  (3 sets: 10 reps/leg/set) | Lunge squat on BOSU ball and inflated balance disc with 5 kg dumbbells  (3 sets: 10 reps/leg/set) |
| Airex® Balance-pad Elite exercise | Single-leg squat with balance-pad  (3 sets: 10 reps/leg/set) | Single-leg standing with balance-pad and the non-supporting leg stretches  backward  (3 sets: 12 reps/leg/sets) | Single-leg support with  balance-pad elite and the non-supporting leg stretches  backward with elastic band  (3 sets: 12 reps/leg/sets) |
| Rest | Between exercise: 60 s Between sets: 3 min | | |

Note: CT group conducted training program on unstable support (e.g., BOSU ball, Swiss ball, and Balance pad); and PT group conducted training program on stable support (i.e., solid floor).

**Table S2.** The plyometric training (PT) program for CT and PT group.

| Exercises | The first stage (1-2 weeks) | The second stage (3-4 weeks) | The third stage (5-6 weeks) |
| --- | --- | --- | --- |
| Front barrier jump (6 hurdles) | Double-leg front barrier jump (15 cm)  (3 sets: 10 reps/set) | Single-leg front barrier jump (15 cm)  (3 sets: 5 reps/leg/set) | Single-leg front barrier jump (30 cm)  (4 sets: 5 reps/leg/set) |
| Lateral high-knees with hurdles | 4-hurdle (15 cm)  (3 sets: 2 reps/set) | 6-hurdle (30 cm)  (3 sets: 4 reps/set) | 6-hurdle (30 cm)  (3 sets: 6 reps/set) |
| Lateral barrier jump | Double-leg jump (15 cm)  (3 sets: 10 reps/set) | Double-leg jump (30 cm)  (3 sets: 12 reps/set) | Single-leg jump (30 cm)  (3 sets: 15 reps/leg/set) |
| Depth jump | Jump with 20 cm box  (3 sets: 8 reps/set) | Jump with 30 cm box  (3 sets: 8 reps/set) | Jump with 40 cm box  (3 sets: 8 reps/set) |
| Multi-direction jumps with hurdles | Triangle jump with double-leg (3 hurdles)  (3 sets: 6*3 reps/set) | Square jump with single-leg (4 hurdles)  (3 sets: 8*3 reps/set) | Hexagon jump with single-leg (6 hurdles)  (3 sets: 12*3 reps/set) |
| Intensity and number of contacts with ground | Low intensity  144 | Middle intensity  234 | High intensity  325 |
| Rest | Between exercise: 60 s Between sets: 3 min | | |
